# Pregnancy Vaccination with Gold Glyco-Nanoparticles Carrying *Listeria monocytogenes* Peptides Protects against Listeriosis and Brain- and Cutaneous-Associated Morbidities

**DOI:** 10.3390/nano6080151

**Published:** 2016-08-19

**Authors:** Ricardo Calderón-Gonzalez, Héctor Terán-Navarro, Elisabet Frande-Cabanes, Eva Ferrández-Fernández, Javier Freire, Soledad Penadés, Marco Marradi, Isabel García, Javier Gomez-Román, Sonsoles Yañez-Díaz, Carmen Álvarez-Domínguez

**Affiliations:** 1 Grupo de Nanovacunas y Vaculas Celulares Basadas en Listeria y Sus Aplicaciones en Biomedicina, Instituto de Investigación Marqués de Valdecilla, Avda. Cardenal Herrera Oria S/N, 39011 Santander, Cantabria, Spain; ricardocalderongonzalez@hotmail.com (R.C.-G.); hteran35@hotmail.com (H.T.-N.); desli87@hotmail.com (E.F.-C.); ferrandez.eva@gmail.com (E.F.-F.); 2 Servicio de Anatomía Patológica, Hospital Universitario Marqués de Valdecilla, Avda. Valdecilla 25, 39008 Santander, Cantabria, Spain; javifreiresalinas@gmail.com (J.F.); apagrj@humv.es (J.G.-R.); 3 CIC-biomaGUNE. P de Miramon, 20009 San Sebastian, Gipuzcoa, Spain; spenades@cicbiomagune.es (S.P.); marcomarradi76@gmail.com (M.M.); igarcia.ciber-bbn@cicbiomagune.es (I.G.); 4 Biomedical Research Networking Center in Bioingeneering, Nanomaterials and Nanomedine (CIBER-BBN), P de Miramon 182, 20009 San Sebastian, Gipuzkoa, Spain; 5 Servicio de Dermatología, Hospital Universitario Marqués de Valdecilla, Avda. Valdecilla 25, 39008 Santander, Cantabria, Spain; syanez@humv.es

**Keywords:** glyconanoparticles, listeria peptides, vaccines, melanocytes

## Abstract

Listeriosis is a fatal infection for fetuses and newborns with two clinical main morbidities in the neonatal period, meningitis and diffused cutaneous lesions. In this study, we vaccinated pregnant females with two gold glyconanoparticles (GNP) loaded with two peptides, listeriolysin peptide 91–99 (LLO_91–99_) or glyceraldehyde-3-phosphate dehydrogenase 1–22 peptide (GAPDH_1–22_). Neonates born to vaccinated mothers were free of bacteria and healthy, while non-vaccinated mice presented clear brain affections and cutaneous diminishment of melanocytes. Therefore, these nanoparticle vaccines are effective measures to offer pregnant mothers at high risk of listeriosis interesting therapies that cross the placenta.

## 1. Introduction

Human listeriosis is a food-borne invasive illness caused by the pathogen *Listeria monocytogenes* (LM) that causes infections in pregnant women, infants, the elderly, and the immune-compromised. While infections are rare among the healthy population, listeriosis is one of the most lethal bacterial diseases for fetuses and newborns [1,2,3,4,5,6,7]. Nevertheless, pregnant women who get infected experience only mild symptoms, making the diagnosis very difficult, even if fetuses are fatally infected [1]. Therefore, vaccination of pregnant women appears as the most cost-effective measure to deal with this deadly pathogen in pregnancy, as it may be responsible for a high number of non-diagnosed spontaneous abortions.

Our group has been preparing vaccines for this pathogen in different formats. We have successfully developed dendritic cell vaccines loaded with LM peptides and deciphered that two peptides from this pathogen’s virulence factors, listeriolysin O (LLO) and glyceraldehyde-3-phosphate-dehydrogenase (GAPDH), LLO_91–99_ and GAPDH_1–22_, conferred significant listeriosis protection in two mice models sensitive and resistant to listeriosis [8,9,10]. We have also prepared a gold glyconanoparticle (GNP) coupled to the LLO_91–99_ peptide formulated with an adjuvant and achieved good vaccine effectiveness in adult mice [11]. Indeed, the application of nanotechnology in immunology is creating big expectations in the field of vaccination [12] and many examples of nanoparticles (NP) have appeared in the literature [13,14]. Nanoparticle-based vaccine candidates have been developed in order to improve the adjuvant effect of the vaccine formulations [15,16], to deliver immunologically active components to target sites [17] and to trigger immune-modulation of inflammatory responses depending on the mechanisms of NP uptake and interactions with immune cells [18]. Among synthetic NPs, GNPs have recently been reported to be able to potentiate the adaptive immune response towards carbohydrate antigens based on the concept of multivalency and depending on the antigen type and loading [19,20,21]. In fact, nanoparticles are promising vector systems to explore vaccination during pregnancy against infectious agents [22]. The interesting results obtained with GNP-LLO_91–99_ [11] prompted us to prepare other glyconanoparticles, GNP-GAPDH_1–22_, and examine the ability of these two vaccine vectors, GNP-LLO_91–99_ and GNP-GAPDH_1–22_, to protect fetuses from listeriosis. For this purpose, we vaccinated pregnant mothers with the above-mentioned glyconanoparticles formulated with adjuvants and followed LM infection during pregnancy and the neonatal period as well as the morbidities associated with neonatal listeriosis.

## 2. Results

### 2.1. Nanoparticle Vaccine Effectiveness in Pregnancy Amilorates Listeriosis-Associated Morbidities

**Figure 1:**
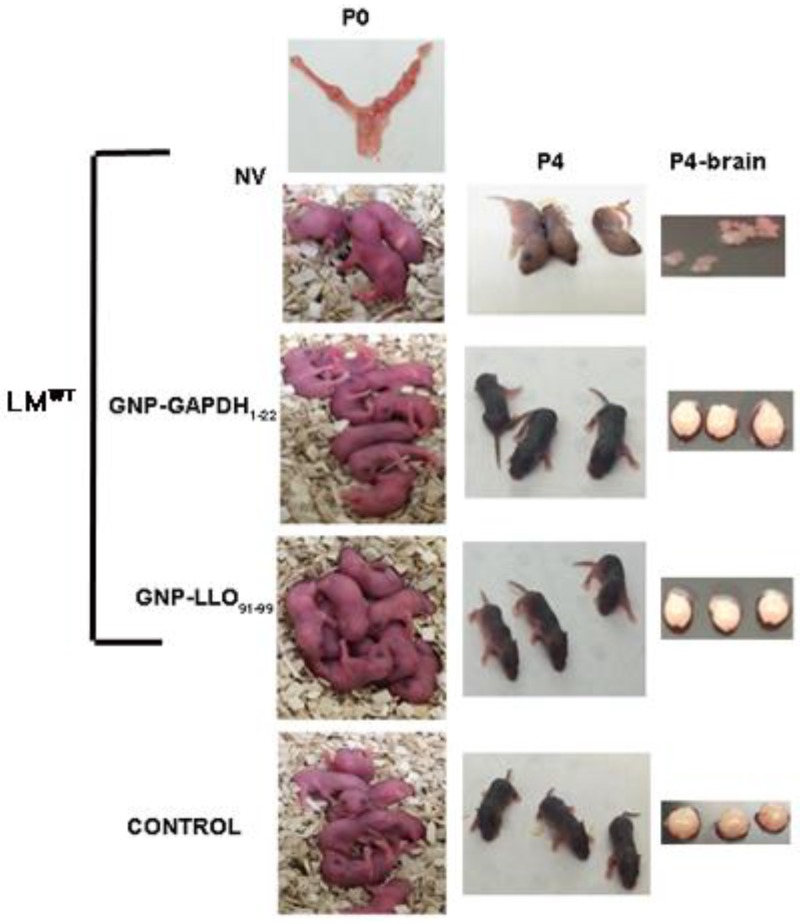
P0 corresponds to day 1 and P4 to day 4 of pups born to pregnant mothers. Groups of pregnant mothers were the following: non-vaccinated and challenged with *Listeria monocytogenes* (LM^WT^) (NV), vaccinated and challenged with LM^WT^ (gold glyconanoparticles listeriolysin peptide 91–99 (GNP-LLO_91–99_) or gold glyconanoparticles glyceraldehyde-3-phosphate dehydrogenase 1–22 peptide (GNP-GAPDH_1–22_)) or non-vaccinated and non-infected with LM^WT^ (control). P4-brain corresponds to brains of P4 neonates.

Neonatal listeriosis is characterized by at least three morbidities in newborns: stillbirths, central nervous sytem affection and cutaneous diffused lesions [23,24]. We vaccinated pregnant mothers of C57BL/6 mice (n = 2) at day 9 of gestation (E9) with two prepared nanoparticle vaccines, GNP-LLO_91–99_ and GNP-GAPDH_1–22_, formulated with Advax^TM^ adjuvant, followed by three days of challenge with LM^WT^ on day 16 of gestation (E16). We prepared three groups of pregnant mothers (n = 2), vaccinated as above, non-vaccinated but challenged with LM^WT^ (NV) and non-vaccinanted and non-infected (control). Control mothers non-vaccinated and non-infected gave birth to nine pups (lower left P0 images in Figure 1), a normal number in C56BL/6 mice that usually deliver six to nine pups. Similarly, GNP-GAPDH_1–22_- or GNP-LLO_91–99_-vaccinated and LM^WT^-challenged mothers delivered nomal numbers of pups, nine and eight, respectively (middle left P0 images in Figure 1). However, NV- and LM^WT^-challenged mothers only delivered two to three pups (upper P0 images in Figure 1). The reduction of neonates in these groups of NV mothers was due to stillbirths or resorbed fetuses as we detected in the mother’s uterus (upper left P0 image in Figure 1). On day 4 after birth (P4), all pups were explored for clinical data, weight, length, coordination movement tests and general and skin observations with a magnifying lens. Clinical data (Table 1) were normal in the control group and GNP-GAPDH_1–22_- and GNP-LLO_91–99_-vaccinated mothers, but they were clearly impaired in the group of NV mothers. The NV group of pups showed weight loss, no ability to move in the metric paper test and a light grey color and wrinkled skin compared to the black and normal skins of vaccinated and control groups (Table 1 and NV images under P4 in Figure 1).

These results suggested that pups born to NV mothers showed central nervous system retardation and enlarged heads and cutaneous immaturation. To confirm these morbidites we sacrificed P4 neonates from the four groups of pregnant mothers. Brains were collected and examined (P4-brain images in Figure 1) and we detected softer cranial covers in pups born to NV mothers (NV images under P4-brain in Figure 1), a significant reduction of blood vessels confirmed by immunohistochemical analysis and a reduced number of melanocytes in the skin with strong apoptotic staining (Table 2).

### 2.2. Nanoparticles Vaccine Reduced the Number of Viable Bacteria in Microglia and Pro-Inflammatory Cytokine Production

**Figure 2:**
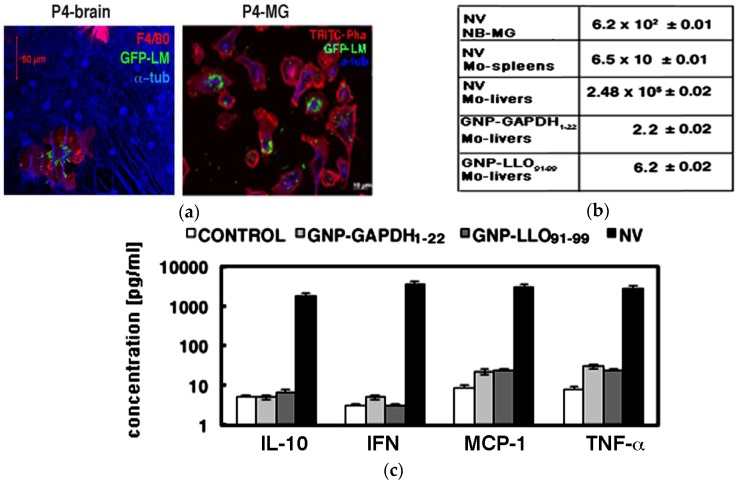
(**a**) P4-brain image corresponds to mixed microglía (MG) cultures of cerebellum brains showing green fluorescence of green fluorescence protein *Listeria monocytogenes* (GFP-LM), red fluorescence of the macrophage marker F4/80 and blue fluoresecence of neuron tubuline. P4-MG corresponds to purified MG of mixed cultures showing green fluorescence of GFP-LM, red fluorescence of actin cytoskeleton stained with TRITC-phalloidin and blue fluorescence of tubuline; (**b**) colony-forming-units (CFU)/mL in MG of newborn P4 pups (NB) born to NV mothers, GNP-LLO_91–99_ or GNP-GAPDH_1–22_ vaccinated mothers. CFU/mL in livers and spleens of NV mothers are also examined; (**c**) Cytokines levels (pg/mL) in supernatants of MG of newborn P4 pups born to mothers as in Figure 1.

We observed that P4 pups born to NV mothers infected with fluorescent green fluorescence protein *Listeria monocytogenes* (GFP-LM^WT^) presented high microglía (MG) infection (green fluorescence in P4-brain, in panel a, Figure 2). MG were characterized by the macrophage F4/80 marker (red fluorescence in P4-brain, panel a, Figure 2). However, we did not observed any bacteria in other brain cells such as neurons as detected with the anti-a-tubuline antibody (blue fluoresnce in P4-brain, panel a, Figure 2), similar to results of in vitro infection of mixed MG cultures [25]. Moreover, purified and isolated MG of P4 pups born to NV mothers presented a severe infection using GFP-LM^WT^ (green fluorescence in P4-MG images, panel a, Figure 2). Numbers of colony-forming-units (CFU) in P4 ewborn pups (NB) born to NV mothers confirmed a high number of viable bacteria, with 6.2 × 10^2^ CFU/mL detected in MG. We detected very few bacteria in MG of P4 pups born to GNP-LLO_91–99_- and GNP-GADPH_1–22_-vaccinated mothers, with 6.2 and 2.2 CFU/mL, respectively (panel b, Figure 2). Next, we also explored listeriosis in the mothers and observed no viable CFU in livers or spleens from GNP-LLO_91–99_- or GNP-GAPDH_1–22_-vaccinated mothers (data not shown). However, we detected 2.40 × 10^5^ CFU/mL in livers of NV mothers that corresponded with 25-fold growth of bacteria compared to initial inoculation with 10^4^ CFU/mice (panel b, Figure 2) and suggested a normal listeriosis infection in these NV mothers. The low CFU in spleens of NV mothers, 6.5 × 10 CFU/mL, indicated these pregnant mothers cleared the infection, while placental transmission was fatal for their fetuses, and after four to six days all pups died (data not shown).

### 2.3. Nanoparticle Vaccines Shifted a Th2 Cytokine Pattern to Th1 Production

Cerebral listeriosis in adults is characterized by high levels of Th1 (TNF-a and MCP-1) and Th2 cytokines (IL-6, IL-10) [24,26,27,28]. Vaccination of systemic listeriosis reduced IL-6 levels (Th2 pattern) and presented a significant increase in IL-12 levels (Th1 pattern) [11]. Here, we confirmed that neonatal listeriosis also caused MG to produce high levels of TNF-a (panel c, Figure 2) and IL-6 (Table 3), as reported in most neonatal human meningitis [1,29]. Vaccination of pregant mothers with GNP-LLO_91–99_ and GNP-GAPDH_1–22_ vaccines caused a signficant increase of IL-12, and a decrease of IL-6 levels (Table 3).

## 3. Discussion

The objective of this study was to identify an optimal vaccine formulation to protect against neonatal listeriosis. Preconceptual vaccination with attenuated *L. monocytogenes* strains does not protect against fetal wastage or placental-fetal invasion [30], prior to or during pregnancy. Therefore, different vaccination vectors or systems should be used to protect against infections of vertical transmission. In this regard, dendritic vaccines emerged as effective vectors able to confer significant protection against systemic listeriosis in different mice models, either sensitive or more resistant to LM infection [8,9,23]. Further, dendritic vaccines loaded with peptides from virulence factors of LM such as lysteriolysin O (LLO) or glyceraldehyde-3-phosphate dehydrogenase (GAPDH) were effective for protection, but they are expensive measures to offer all pregnant women at risk of listeriosis. In this regard, nanoparticles are promising vaccine vectors with interesting properties such as safety, biocompatibility, and versatility to load with different ligands and directionality to dendritic cells with chemical modifications such as carbohydrates [31,32,33].

We previously reported that gold glyconanoparticles loaded with a listeriolysin O peptide, GNP-LLO_91–99_, when formulated with adjuvants such as Advax^TM^, conferred significant protection against systemic listeriosis in adult animal models [11,23]. Taking advantage of this nanoparticle system, we also designed a similar vector coupled with another peptide of the GAPDH virulence factor, GAPDH_1–22_, which showed promising properties in dendritic cell vaccines [8,9]. Here, we presented that both GNP-LLO_91–99_ and GNP-GAPDH_1–22_, nanovaccines formulated with Advax^T004D^ conferred good protection during pregnancy against neonatal listeriosis. GNP-GAPDH_1–22_ nanovaccines appeared more effective than GNP-LLO_91–99_ nanovaccines as they showed lower CFU numbers in MG. Moreover, these nanovaccines reduced central nervous system (CNS) and skin-associated morbidities as well as pregnancy complications.

Invasive listeriosis in pregnancy is a fatal infection for the fetuses, causing complications such as spontaneous abortions or stillbirths in 20% of the cases, especially if infection occurs early in the pregnancy; preterm delivery and neonatal infection are also observed [1,34]. 70% of surviving neonates born to mothers infected with LM, presented trans-placental transmission of bacteria. Neonatal listeriosis manifestations imply meningitis, brain abscesses, diffused skin lesions and rash, fever and lethargy [1,2,3,4,5,6,7,23,26]. While not exactly similar, neonatal experimental listeriosis inoculating pregnant mice with pathogenic LM reproduces most of the clinical symptoms [26,27,28,34]. In this regard, our results here detecting resorbed fetuses in mice mothers might reflect human stillbirths; the 2- to 3-fold reduction in neonates at birth might mimic spontaneous abortions, all clinical complications related to pregnancy [1]. We also detected CNS-associated morbidities such as enlarged heads, softer cranial covers and a lack of coordinated movement that resembled CNS impairment [24]. Finally, the lower number of skin melanocytes due to apoptosis we detected in P4 pups born to NV mothers appeared comparable to cutaneous diffused lesions of granulomatosis infantiseptica forms of severe listeriosis in neonates [23]. All these above-mentioned listeriosis-associated morbidities disappeared in pregnant mothers pre-vaccinated with GNP-GAPDH_1–22_ and GNP-LLO_91–99_ nanovaccines and in neonates born to these vaccinated mothers. Therefore, they highlighted the ability of these nanovaccines to cross the plancental barrier and protect the fetuses.

Moreover, the ability of these nanovaccines to shift the cytokine pattern of MG [35] towards a Th1-IL-12-dependent pattern is supposed a good benefit since IL-12 production seems associated with vaccine efficiency and long-term protection [8,11,28], but also because acute Th2 cytokines such as IL-6 [29] were significantly reduced after vaccination with GNP-GAPDH_1–22_ and GNP-LLO_91–99_ nanovaccines.

We concluded that GNP-LLO_91–99_ and GNP-GAPDH_1–22_ nanovaccines are effective measures for vaccination against listeriosis during pregnancy, with GNP-GAPDH_1–22_ being more potent than GNP-LLO_91–99_ nanovaccines. The disappearance of listeriosis morbidities is an added value of these nanovaccines that revealed their powerful action.

## 4. Materials and Methods

Nanoparticles: GNP carrying approximately 90% glucose and 10% LLO_91–99_ peptide were prepared by reduction in situ of Au(III) salt with sodium borohydride (Figure S1, panel a and b) [11], following previously described procedures [30,31,35,36]. To obtain GNPs carrying GAPDH peptide, an aqueous solution of tetrachloroauric (Strem Chemicals) (0.025 M, 1 eq.) was added to a solution of a mixture of glucose (90%) and GAPDH peptide (10%) thiol ending ligands (0.012 M, 6 eq.) in MeOH/H_2_O/CH_3_COOH (3:3:1). An aqueous solution of NaBH_4_ (1 M, 22 eq.) was then portion-wise added and the mixture was shaken for 2 h at 25 °C. The solvent was evaporated at reduced pressure. The residue was washed with ethanol, re-dissolved in the minimum quantity of milliQ water, loaded into 5–10 cm segments of SnakeSkin^®^ pleated dialysis tubing (Pierce, 3500 MWCO) and purified by dialysis against distilled water (3 L of water, recharging with fresh water every six hours over the course of 72 h). The nanoparticles were obtained as brown powder after lyophilization (Figure S1, panel b shows prepared GNP-LLO_91–99_ and GNP-GAPDH_1–22_). The size distribution of the gold nanoparticles was evaluated from several transmission electron microscopy (TEM) micrographs (JEM-2100F, JEOL Ltd., Tokyo, Japan). TEM (average diameter and number of gold atoms): 2.1 ± 0.5 nm (Figure S1, panel c). The presence of glucose and peptide ligands was confirmed by ^1^H NMR (500 MHz, D_2_O): 7.3 (br s 4.4 ~5H), 4.4 (br s, ~1H), 4.1–3.1 (br m, ~57H), 2.6 (t, ~3H), 2.5 (t, ~3H), 1.99–1.15 (br m, ~37H), 0.87 (br s ~22H) (Figure S2, panel d). The amount of GAPDH peptide on the GNPs was determined by quantitative NMR (qNMR) in a Bruker AVANCE 500 MHz spectrometer (Bruker Corp., Billerica, MA, USA): 0.234 mg of GNP were dispersed in D_2_O 99.9% (200 μL). Then 80 μL of this solution were added with 40 μL of a 0.05% 3-(trimethylsilyl)propionic-2,2,3,3-d4 acid sodium salt (TSP-d4) solution in D_2_O and 60 μL of D_2_O. Peptide loading was 10.6 μg/0.234 mg GNPs. GNP-LLO_91–99_ and GNP-GAPDH_1–22_ stability was performed after following dendritic cells incubation for 8 to 24 h in vitro and FACS analysis of peptides using anti-LLO (Diatheva) and anti-GAPDH_1–22_-specific antibodies made in rabbit [8]. We observed 90% and 95% double positive CD11c^+^anti-LLO^+^ and CD11c^+^anti-GAPDH_1–22_^+^ in DC recovered cells after treatment for 24 h with GNP-LLO_91–99_ or GNP-GAPDH_1–22_, respectively.

Bacteria. Daniel A. Portnoy (University of California, Berkeley, CA, USA) provided *L. monocytogenes* 10403S pathogenic strain (LM^WT^) and the mutant strains LM^∆LLO^ (DPL-2161) and LM^∆ActA^ (DPL-1942). GFP-*LM* DH-L1039 (GFP-LM) derived from the 10403S LM strain was a gift from Darren E. Higgins (Harvard Medical School, Boston, MA, USA).

Animals. We used C57BL/6 mice from our animal facilities at the University of Cantabria at 8–12 weeks old. Three female and one male were mated and assessed for the appearance of vaginal plug denoting first embryonic day of pregnancy.

Pre-natal vaccination. Pregnant C57BL/6 female mice were vaccinated or not (NV) at day 9 of gestation (E9) with intravenously (*i.v*) via the lateral tail vain with GNP-LLO_91–99_ or GNP-GAPDH_1–22_ formulated with Advax^TM^ (5 µg of nanoparticles and 250 µg of adjuvants/mice). Next, at 16 days of gestation (E16) all mice, vaccinated and NV were inoculated *i.v* with 100 µL of a LM^WT^ bacterial suspension in saline (1 × 10^5^ CFU/mL) (LM^WT^ infected mothers). We also preserved a group of mothers NV and inoculated with 100 µL of saline (Control). All animals were daily examined. At E20 we detected two to three pups born to LM^WT^ infected mothers and NV, while six to eight pups born to control and vaccinated mice. Four days after birth, P4 postnatal pups born to LM^WT^ infected and vaccinated (GNP-LLO_91–99_ or GNP-GAPDH_1–22_) or NV mothers were sacrificed to obtain cerebellum for preparation of mixed microglia and subsequent isolation of primary microglial cultures for CFU quantification and cytokine quantification. LM^WT^ infected and vaccinated, NV and control mothers were bled for cytokine analysis and sacrificed to obtain uterus for observation of resorbed fetuses and spleens and livers for CFU quantification.

Clinical tests. Clinical test were performed on all pups born to mothers vaccinated, NV or control on days P0 and P4. Test include weight, length, coordination-movement assays placing pups in a metric paper and recording the centimeters of movement after 5 min, general and cutaneous screening for lesions and wounds with a magnifying glass.

Microglia isolation. Microglial cultures have been described [37,38] and detailed procedures for obtaining mixed microglial cultures and purified primary microglia were reported [24]. In brief, mixed microglial cell cultures were obtained from cerebellum at P4 until complete neuronal differentiation for seven days as described [37]. Microglial cultures shaked at 200 times/min for 30 min and cells in supernatants re-plated in 24-well plates. MG cells were 90% double positive CD11b^+^CD45^+^ and 80% double positive for F4/80^+^IA^b+^ as measured by flow cytometry (FACS). Supernatants of MG cells were filtered and stored at −80 °C for cytokine analysis by FACS and next, MG cells were lysed and plated in BHI agar plates for CFU analysis.

Confocal assays. Cells used for confocal microscopy were fixed in 3% paraformaldehyde. Fluorescence labeling and confocal microscopy were performed as previously described [24].

FACS analysis. Cell surface markers of MG were analyzed by FACS using the following antibodies: anti-CD11b-FITC, anti-CD45-PerCP, anti-F4/80-PE and anti-IA^b^-brilliant blue (Miltenyi Biotech Inc., Auburn, CA, USA). Annexin-V conjugated with APC fluorochrome and 7-ADD (7-amino-actinomycin D) (BD Biosciences, San Jose, CA, USA) and cytokines in MG supernatants were also quantified using the CBA kit (BD Biosciences, San Jose, CA, USA). Samples were analyzed in triplicates and results are expressed as the mean ± SD of two separate experiments.

Immunochemistry. One mice (NB) of each group born of vaccinated, NV or control mothers were immersed in 4% formaldehyde for 24 h. Organs were subsequently embedded in paraffin, processed and sections stained with hematoxylin-eosin and immunohistochemical analysis for apoptotic cells (TUNNEL staining performed according to the manufacturer instructions, Roche), and main antibodies characteristic of different organs (brain, skin, liver, gut, spleen, colon, lungs) as well as with anti-*Listeria* antiserum (Difco, BD Biosciences, New York, NY, USA) to localize the bacteria. For skin analysis, sections of 1 mm of skin were examined for melanocytes number and apoptosis.

Statistical analysis. For statistical analysis, the Student’s *t* test was applied. *p* ≤ 0.05 was considered significant.

Ethics statement. This study was carried out in strict accordance with the recommendations in the guide for the Care and Use of Laboratory Animals of the Spanish Ministry of Science, Research and Innovation. The Committee on the Ethics of Animal Experiments of the University of Cantabria approved this protocol (Permit Number: 2012/06) that follows the Spanish legislation (RD 53/2013). All efforts were made to minimize suffering.

## 5. Conclusions

GNP-LLO*_91–99_* and GNP-GAPDH*_1–22_* nanovaccines formulated with Advax adyuvant protect against listeriosis when applied during pregnancy. Nanovaccines are effective measures for preganancy vaccination as they are able to cross the placental barrier and diminish listeriosis morbidities.

## Figures and Tables

**Table 1:** Clinical data of pups (P4) born to vaccinated, non-vaccinated (NV) or control mothers.

	Weight (mg)	Length (cm)	Coordination Movement ^b^ (cm)	Cutaneous Test ^c^
**NV ^a^**	170 ± 0.5	5.0 ± 0.1	0.2 ± 0.1	grey-wrinkled
gold glyconanoparticles listeriolysin peptide 91–99 (**GNP-LLO_91–99_**)	280 ± 0.4	4.0 ± 0.1	9.5 ± 0.8	black-normal
gold glyconanoparticles glyceraldehyde-3-phosphate dehydrogenase 1–22 peptide (**GNP-GAPDH_1–22_**)	295 ± 0.5	4.2 ± 0.1	10 ± 0.7	black-normal
**CONTROL**	300 ± 0.5	4.0 ± 0.1	10 ± 0.8	black-normal

^a^ Four groups of pregnant mothers were prepared: non-vaccinated (NV), GNP-LLO_91–99_-vaccinated, GNP-GAPDH_1–22_-vaccinated and controls (non-vaccinated). NV and vaccinated groups were challenged with *Listeria monocytogenes* (LM^WT^) but not the control group. All P4 pups were examined for clinical data; ^b^ Coordination movement test measures in a 10 cm metric paper the length (cm) achieved by each group of mice in 5 min. Results are the mean ±SD of triplicates; ^c^ Cutaneous test with magnifying lens to confirm skin color (black, grey or white) and aspect (normal or wrinkled).

**Table 2:** Number of relative melanocytes and blood vessels.

	Relative Melanocytes ^a^	Apoptotic Melanocytes ^b^ (%)	Blood Vessels ^c^
**NV**	0.2 ± 0.1	70 ± 0.8	Few
**GNP-LLO_91–99_**	1 ± 0.1	1 ± 0.1	Normal
**GNP-GAPDH_1–22_**	1 ± 0.1	0.9 ± 0.1	Normal
**CONTROL**	1 ± 0.1	0.9 ± 0.1	Normal

^a^ Relative melanocytes, correspond to the number of melanocytes per 1 mm of skin measured by immunohistochemistry; ^b^ Apototic melanocytes, correspond to the percentages of melanocytes positive for the TUNNEL staining; ^c^ Blood vessels were observed in whole brains with magnifying lens and classified as few or normal.

**Table 3:** Cytokine levels of purified microglía (MG) from P4 neonates born to vaccinated or NV mothers.

	IL6 (pg/mL) ^a^	IL-12 (pg/mL)
**NV**	1650 ± 0.9	17 ± 0.8
**GNP-LLO_91–99_**	37 ± 0.1	27 ± 0.1
**GNP-GAPDH_1–22_**	55 ± 0.1	40 ± 0.1
**CONTROL**	3 ± 0.1	1 ± 0.1

^a^ Cytokines are measured by flow cytometry and results expressed as concentration of pg/mL ± SD of triplicates.

## Supplementary Materials

The following are available online at www.mdpi.com/2079-4991/6/8/151/s1.

## Abbreviations

The following abbreviations are used in this manuscript:

GFP: green fluorescence protein
CFU: colony forming unit
CNS: central nervous system
GAPDH: glyceraldehyde-3-phosphate-dehydrogenase
GNP: glyconanoparticle
IFN: interferon
*i.v*: intravenously
LLO: listeriolysin O
LM: *Listeria monocytogenes*
