# Supplementary material for: Pregnancy Vaccination with Gold Glyco-Nanoparticles Carrying Listeria monocytogenes Peptides Protects against Listeriosis and Brain- and Cutaneous-Associated Morbidities
Source: Nanomaterials (Basel). 2016 Aug 19;6(8):151. doi: 10.3390/nano6080151 (PMC5224619; doi:10.3390/nano6080151)
Supplement: Supplementary file 1 [file nanomaterials-06-00151-s001.pdf]

Supplementary Materials: Pregnancy Vaccination with Gold Glyco-Nanoparticles Carrying *Listeria monocytogenes* Peptides Protects against Listeriosis and Brain- and Cutaneous-Associated Morbidities

Ricardo Calderón-Gonzalez, Héctor Terán-Navarro, Elisabet Frande-Cabanes, Eva Ferrández-Fernández, Javier Freire, Soledad Penadés, Marco Marradi, Isabel García, Javier Gomez-Román, Sonsoles Yañez-Díaz and Carmen Álvarez-Domínguez

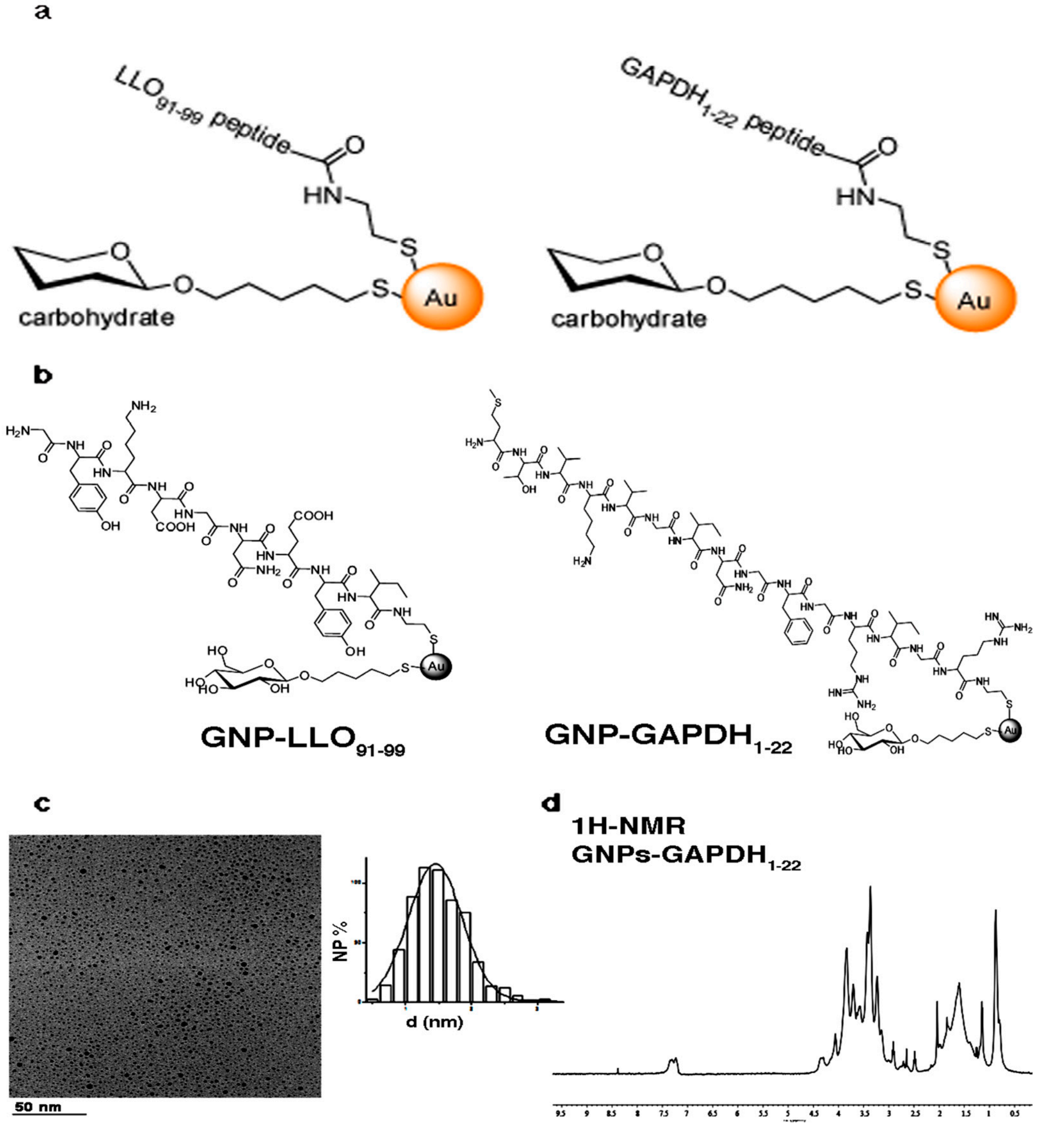

Figure S1. Synthesis of gold glyconanoparticles listeriolysin peptide 91–99 (GNP-LLO*91–99*) and gold glyconanoparticles glyceraldehyde-3-phosphate dehydrogenase 1–22 peptide (GNP-GAPDH*1–22*) nanovaccines. (**a**) Model of nanovaccines used, GNP-LLO*91–99* and GNP-GAPDH*1–22*. (**b**) Schematic representation of chemical structure of gold glyconanoparticles coated with LLO (GNP-LLO*91–99*) or GAPDH (GNP-GAPDH*1–22*) peptides. (**c**) Transmission electron microscopy (TEM) image (100,000 × magnification) and size histogram showing the spherical shape and nanometric size of the gold core of GNP-GAPDH*1–22*. (**d**) 1H-NMR analysis of GNP-GAPDH*1–22* to calculate the amount of peptide: 8.9 µg (GAPDH*1–22*)/0.182 mg of GNP.
